# Bone response to intermittent parathyroid hormone (PTH) is both genetic and sex specific

**DOI:** 10.64898/2026.06.09.729629

**Published:** 2026-06-10

**Authors:** Douglas J. Adams, Dana A. Godfrey, Shane E. Ridoux, Robert D. Maynard, Nicole S. Szeto, Cheryl L. Ackert-Bicknell

**Affiliations:** 1 Colorado Program for Musculoskeletal Research, Department of Orthopedics, University of Colorado School of Medicine, Aurora, CO; 2 Department of Biomedical Informatics, University of Colorado, Aurora, CO; 3 Department of Mathematical and Statistical Sciences, University of Colorado, Denver, CO

## Abstract

Teriparatide (PTH 1–34) is an anabolic agent used to treat osteoporosis, yet clinical response varies widely among patients. To investigate genetic and sex-specific determinants of skeletal response, we administered intermittent PTH to male and female mice from eight genetically diverse inbred strains. Mice were treated for four weeks, and bone phenotypes were assessed via DXA, microCT, and mechanical testing. Response to PTH was highly strain- and sex-dependent, with some strains responding at the femur but not the spine, and vice versa. Heritability estimates for PTH-induced changes in bone mineral density (BMD), cortical area, breaking strength, and trabecular bone volume fraction (BV/TV) ranged from moderate to high, with BV/TV showing the strongest genetic influence. Cortical bone response mechanisms differed by sex: males exhibited periosteal expansion, while females showed endosteal remodeling. These findings mirror clinical observations where hip non-response is more prevalent than spine non-response and suggest that genetic background and sex significantly influence therapeutic outcomes. Our data support the use of genetically diverse mouse models to elucidate the genetic architecture of PTH response and highlight the potential for personalized approaches in osteoporosis treatment. Future genome-wide association studies in outbred mice may identify specific loci mediating skeletal responsiveness to PTH, advancing precision medicine strategies for bone anabolic therapies.

## LAY SUMMARY

Teriparatide, a drug used to treat osteoporosis, consists of the active portion of parathyroid hormone (PTH). Information from clinical studies suggests that not all patients will respond to this medication. We used eight strains of inbred mice to study the impact of genetic background and sex on the response to PTH. We learned that response to PTH is driven by both genetics and sex. Some strains responded at the femur, but not the spine and vice versa. These results may explain why a failure to respond at the hip in humans is more common than at the spine.

## INTRODUCTION

Teriparatide is an anabolic agent used for the treatment of both primary osteoporosis in men and women^(1)^ and secondary osteoporosis due to glucocorticoid use ^(2,3)^. It is a recombinant peptide consisting of the first 34 amino acids of parathyroid hormone (PTH)^(1)^ and like native PTH, teriparatide can stimulate bone formation by osteoblasts and resorption by osteoclasts^(2)^.Teriparatide is commonly administered once daily such that patients are exposed in an intermittent fashion, leading to an increase in osteoblast number and increased bone mass^(1,2)^. Clinical trials data shows excellent efficacy for the reduction of vertebral fracture and non-vertebral fracture, as well as being an effective agent to increase bone mineral density (BMD) of the spine, hip and whole body ^(2)^. Other anabolic agents currently approved in the US include abaloparatide, which acts via a similar mechanism as teriparatide, and romozosomab, a monoclonal antibody that acts through the WNT signaling pathway^(4)^.

Teriparatide was first approved in 2002 in the US^(5)^ and many studies of efficacy and safety have followed. Mounting evidence has suggested that not all patients respond to teriparatide ^(6–8)^ and while factors such as age and vitamin D status have been shown to contribute to the magnitude of response, a full understanding of why some patients do not respond is lacking^(8,9)^. Modifiable environmental factors impact bone in an interactive manner with genetic factors. Indeed, many such Gene by Environment (G*E) interactions impacting bone have been described for factors such as dietary fat, exercise and vitamin D use^(10)^. Like these common environmental variables, response to teriparatide is also thought to be partially mediated by genetic factors. A small genome wide association study (GWAS) was conducted in humans for the phenotypes of “*response of BMD at either the lumbar spine or hip to teriparatide*” ^(11)^ and allelic differences at the *CXCR4* gene on human Chromosome (Chr) 2 were found to reach statistical significance for both BMD sites. Additional suggestive (not genome wide significant) loci were identified on Chrs 15 and 19. Unlike for the loci on Chr 2 and 19, the locus on Chr 15 appears to only be associated with lumbar spine BMD^(11)^. The heritability of response to teriparatide is not known.

In this study, we treated male and female mice from eight inbred strains with either vehicle or PTH for four weeks to better define the heritability of teriparatide response. We determined that the anatomic site of response to treatment varied widely among the strains and that there were strong sex differences among the strains. Further, we observed that some strains would only respond in the cortical compartment and others would present with trabecular response phenotypes. Our results reflect that which was seen in the human teriparatide response GWAS, but provide valuable insight into the nature of response as we can include phenotypes that cannot be captured in humans.

## MATERIALS AND METHODS

### Animals and PTH treatment.

All studies involving mice were reviewed and approved by the Institutional Animal Care and Use Committee (IACUC) of the University of Colorado (CU), Anschutz Medical Campus (Protocol number: 00902). All mice were fed Teklad Irradiated Global Soy Protein-Free Extruded Rodent Diet (Teklad, Cat no. 2920X) and had *ad lib*. access to food and water. The mice were maintained on a 14hr:10hr light:dark cycle. Mice used in this study were purchased from The Jackson Laboratory and the following 8 strains were used with strain stock numbers provided in parentheses: A/J (#000646 ), C57BL/6J (B6, #000664 ), 129S1/SvImJ (129, #002448), NOD/ShiLtJ (NOD, #001976), NZO/HlLtJ (NZO, #002105), CAST/EiJ (CAST, #000928), PWK/PhJ (PWK, #003715 ), and WSB/EiJ (WSB, #001145 ). For each strain, ~40 mice were purchased, half of which were male. Mice were purchased at or near weaning age and allowed to acclimate to the CU vivarium until the age of 12 weeks. At 12 weeks of age, baseline whole body BMD was measured by DXA and each strain of mice was divided into two equal groups for daily i.p. injection, 5 days per week for 4 weeks, with either vehicle (saline) or PTH:1–34 (40 mg/kg). At 16 weeks of age all mice were euthanized and whole body BMD was measured by DXA. The lumbar spine and left femur were collected, wrapped in saline-soaked gauze, and analyzed as described below. Tissues were stored frozen at −20°C until processing. Some samples were damaged during dissection and could not be used for phenotyping. The exact number of mice per group for each type of measurement is shown in Supplemental Tables 1–6.

### Whole Body BMD via Dual X-ray Absorptiometry (DXA)

Areal bone mineral density (aBMD) and bone mineral content (BMC) were measured for the whole body (without head) and isolated to the lumbar spine (L3-L5) using a calibrated Hologic/Faxitron UltraFocus-DXA X-ray cabinet system. Some images could not be analyzed due to movement artifacts from breathing.

### Mechanical testing via 3-point flexure

Fresh-frozen femurs were thawed and tested in 3-point flexure to measure whole bone structural mechanical properties. Femurs were placed anterior side down on 1 mm diameter supports spaced 10 mm apart, applying midspan loading to failure at 0.1 mm/second with force and displacement data acquired at 10 Hz (TA Instruments 3200, Eden Prairie, MN). Flexural strength was defined as the maximum applied force, with structural stiffness measured within the linear portion of the force-deflection response. The distal half of the femur was then imaged via microCT to quantify indices of trabecular and cortical morphometry.

### Bone architecture

Trabecular bone morphometry was quantified in the distal metaphysis of the left femur and vertebral centrum of the L4 vertebra. Cortical bone morphometry was quantified at mid-diaphysis of the femur. Femurs and vertebrae were imaged in 70% ethanol using X-ray micro-computed tomography, collecting 2000 conebeam projections per revolution at 70 kVp (200μA) and an integration time of 500 msec within a 20 mm diameter field of view (μCT50, Scanco Medical AG, Bassersdorf, Switzerland). Three-dimensional images were reconstructed at 10 μm resolution using standard convolution back projection algorithms with Shepp and Logan filtering and rendered at a discrete voxel density of 1,000,000 voxels/mm^3^ (isometric 10 μm voxels). Bone mineral was calibrated to a discrete-step hydroxyapatite phantom and segmented from marrow and soft tissue in conjunction with a constrained Gaussian filter to reduce noise, applying mineral density thresholds of 550 and 700 mg HA/cm^3^ for trabecular and cortical bone, respectively. Volumetric regions for trabecular bone analysis were selected within the endosteal borders of the femoral metaphysis to include the secondary spongiosa located ~1 mm (~7% of length) from the growth plate and extending 5% of femur length proximally. Likewise, the central 80% of vertebral body height within the L4 vertebra was selected for trabecular analysis. Trabecular morphometric parameters were measured without imposing a presumed structural model to obtain direct measures of trabecular volume fraction (BV/TV), thickness (Tb.Th), number (Tb.N) and spacing (Tb.Sp). Cortical transverse morphometry was averaged within a mid-diaphyseal span of ~5% of femur length to obtain measures of total cross-sectional area (bone + medullary area, mm^2^), cortical bone area (mm^2^), marrow area (mm^2^) and bone area fraction (%).

### Statistical analysis

All statistical analysis was conducted in R version 4.4.2. As there are known differences in bone biology between males and females, all analysis was conducted in a sex separated manner. Comparisons between strains was not the goal of this study and therefore, PTH effects differences were determined by a two-sided unpaired T-Test within as strain.

To test for heritability, a simulated population of potential differences between the PTH treated animals and the saline treated animals within a strain and within a phenotype was created by conducting 10,000 bootstraps of the data, using the *boot* package for R (version 1.3–31). The *ICC* package for R (version 2.4.0) was used to calculate the unadjusted interclass correlation coefficient using strain as the classifier. This value was used to estimate the Broad Sense Heritability (H^2^) as per the method suggested by Philip ***et al*** ^(12)^.

## RESULTS

### Bone mineral density and body composition

**Figure 1.**
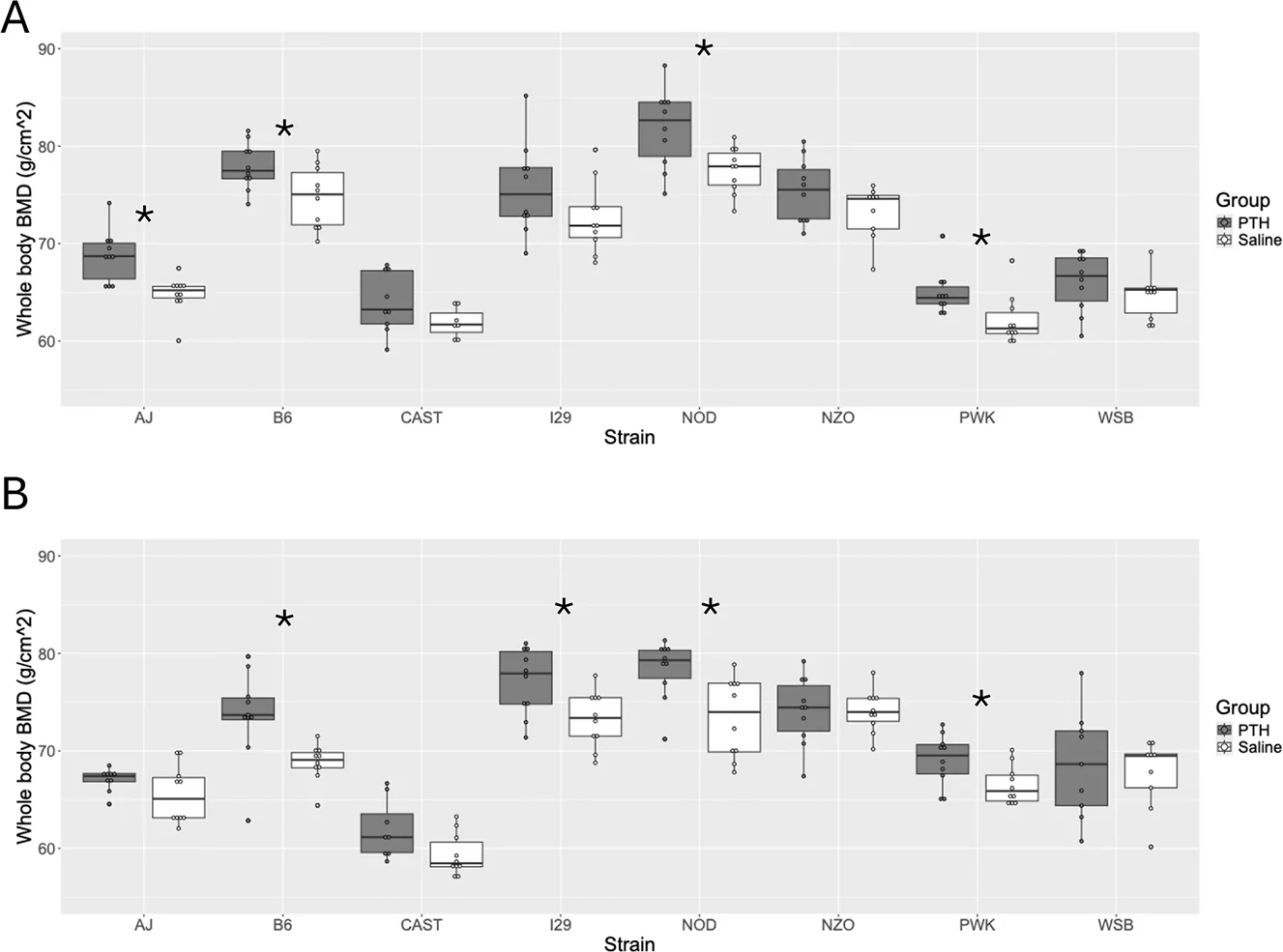
Whole body BMD response to intermittent PTH. Whole body bone mineral density (BMD) sans the head is shown for male (A) and female (B) mice of 8 different inbred strains after 4 weeks of treatment. PTH treated animals are shown in grey and saline treated animals in white. Values for PTH treated animals are shown in grey and saline treated animals in white. *p<0.05.

Administration of PTH did not affect body weight in males or females in any strain (**Supplement Table 1**) and had no effect on lean or fat mass in most strains. As expected, intermittent exposure to PTH increased whole body BMD in B6 male and female mice, the genetic reference strain (Figure 1, **Supplement Table 2**). A robust BMD response was measured in both male and female NOD and PWK mice, but other strains showed sex-specific responses. Specifically, a BMD response was observed in male A/J, but not in female, and a response was seen in female 129 mice but not in male (Figure 1). The broad sense heritability for “BMD response to PTH treatment” was 85.0% for the females and 38.6% for the males (Table 1). This reflects the overall less robust BMD response to PTH observed in the males. The strain-specific response for whole-body bone mineral content (BMC) followed a similar pattern for the males, but only the B6 and NOD strains showed a significant response in the females (**Supplement Table 2A&B**). In the lumbar spine of male mice, only A/J responded to PTH for BMD and BMC. In the lumbar spine of female mice, a positive response for BMD was detected in the CAST, NOD and PWK strains, and for BMC only in the B6 and PWK females (**Supplement Table 2A&B**).

### Femoral Cortical Architecture and strength

**Figure 2.**
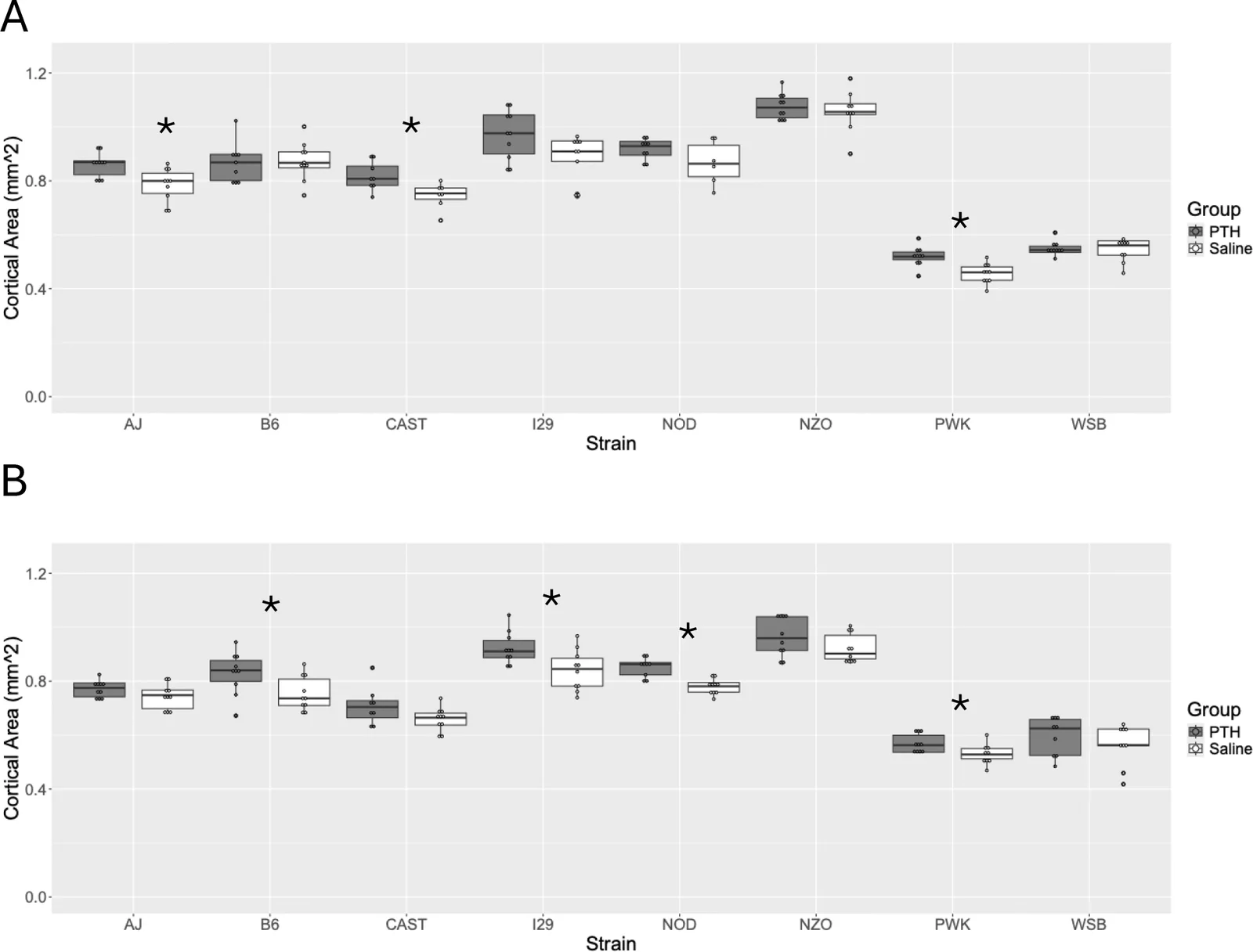
Femoral cortical area response to intermittent PTH. Cortical geometry at the mid diaphysis was measured by μCT after 4 weeks of PTH treatment. Cortical area in PTH treated (grey) and saline treated (white) males (A) and females (B) is present. *p<0.05.

In males, we observed a significant increase with PTH treatment for cortical area in A/J, CAST and PWK mice, and nearly significant increase in the 129 (p=0.0557). Response to PTH was highly heritable for cortical area in males (H^2^ = 71.0%). For all four of these strains there was a concordant increase in cortical thickness. In males, a change in marrow area was detected only in the CAST strain. In females, 129, B6, NOD and PWK mice were observed to have an increase in cortical area (Figure 2, **Supplemental Table 3 A&B**), and this was moderately heritable (H^2^ = 39.7%, Table 1) As occurred in males, cortical thickness was increased in the strains that presented with an increase in cortical area; however, for the PWK strain the increase was just below the significance threshold (p=0.0659). The cortical area response to PTH was less heritable in the females at 39.1% (Table 1). A change in intramedullary area was noted with increased cortical area only in the 129 strain. The A/J and CAST female mice experienced a decrease in intramedullary area and an increase in cortical thickness, but no significant change in cortical area. No change in periosteal circumference was noted for either strain (A/J, PTH = 4.38±0.204 vs. Saline = 4.74±0.244 mm, p = 0.999, CAST, PTH = 3.88±0.193 vs. Saline = 4.01±0.216 mm, p = 0.910).

**Figure 3.**
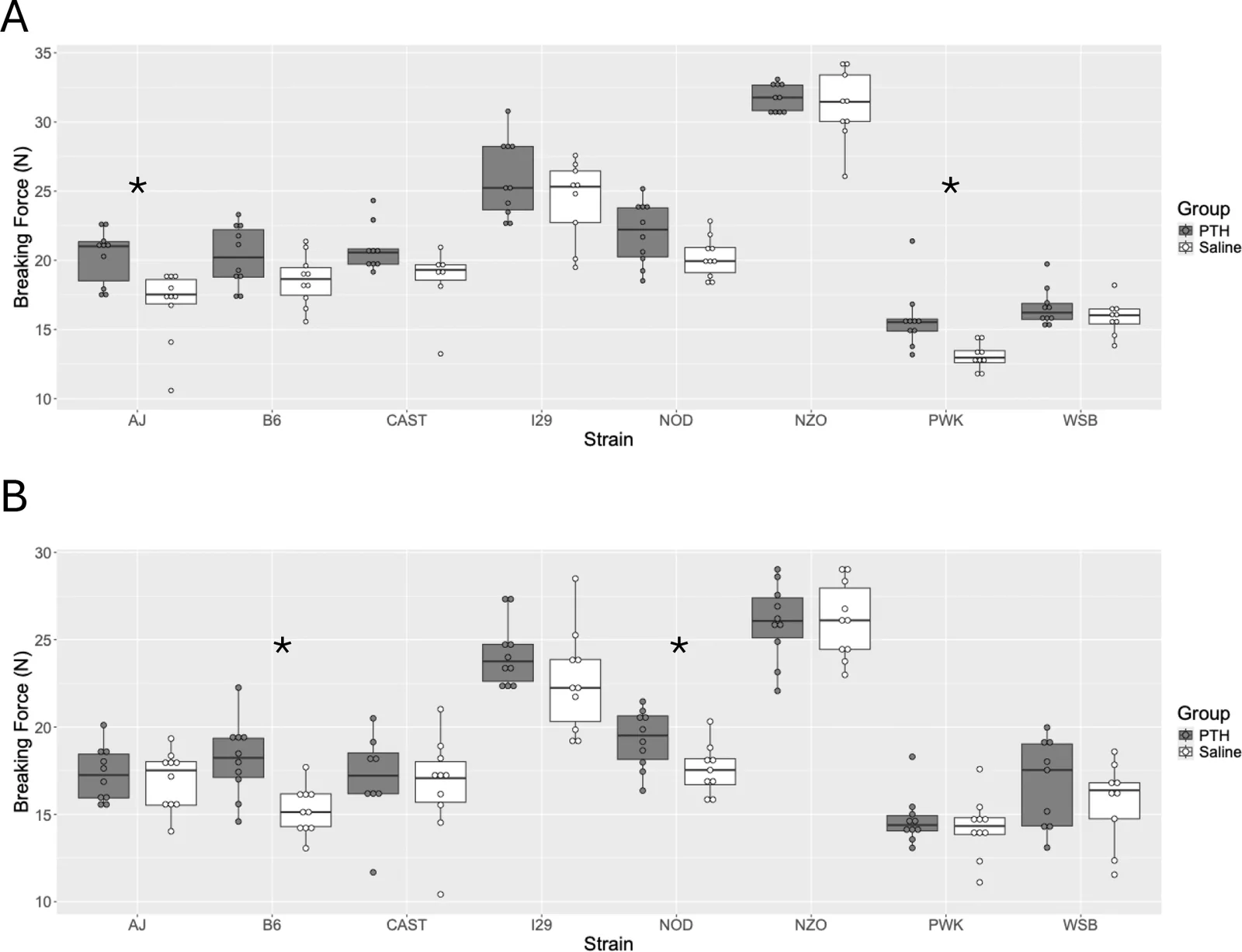
Femoral Breaking Strength in saline and PTH treated mice. Femoral breaking strength was measured by 3-point bending in female (A) and male (B) of 8 inbred strains. Values for PTH treated animals are shown in grey and saline treated animals in white. *p<0.05.

Increases in breaking strength with PTH treatment were seen in male A/J and PWK, and in female B6 and NOD mice (Figure 3, **Supplemental Table 4 A&B**). The heritability for change in breaking strength after PTH treatment was moderate for both males and females (Table 1). This may reflect the high variance in this phenotype. For all four of these groups, there was a concordant increase in cortical area. A change in stiffness was observed only in the NOD males, which did not exhibit a change in breaking strength with PTH treatment, and in B6 females, which did. PTH did not affect work-to-failure in any of the groups. Flexural and torsional moments of area, geometrical measures of strength and stiffness, largely followed the pattern of changes in cortical area.

### Femoral and vertebral trabecular compartment

**Figure 4.**
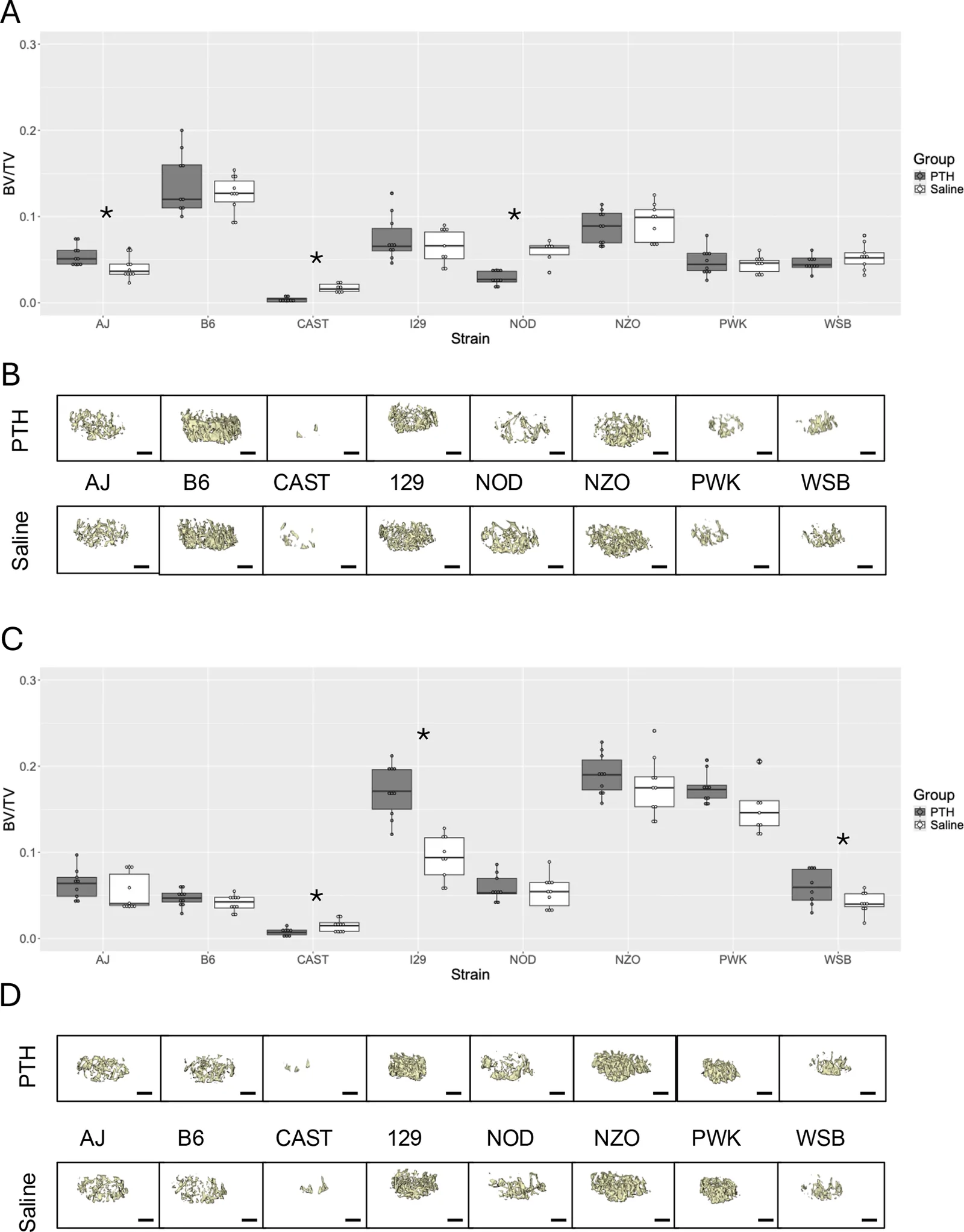
Distal femoral trabecular bone volume fraction (BV/TV) in saline and PTH treated mice. Distal femoral volume fraction (BV/TV) was measured by μCT after 4 weeks of PTH treatment. Date from the PTH treated (grey) and saline treated (white) males (A) and females (C) is present. Representative reconstructions of the trabecular compartment are shown for males (B) and females (D) such that the PTH treated animals are on the top row and the saline treated are on the bottom row. *p<0.05.

In male mice we observed an increase in femoral BV/TV with PTH in AJ, but a decrease in CAST and NODs (Figure 4A and **Supplemental Table 5A)**. In the A/J mice, this increase in femoral trabecular volume fraction was coincident with thicker trabeculae and a large increase in trabecular number. In the CAST males, there was a trend towards thinner trabeculae, but valid measurements of trabecular number could not be performed for the very sparse trabecular volume (Figure 4). The mechanism of decrease could therefore not be determined. In the NOD males, this decrease in BV/TV was associated with a profound reduction in the number of trabeculae, but unlike in the CAST males, the NOD males experienced no change in trabecular thickness. In the B6 and NZO males, we observed a large increase in trabecular thickness that was coincident with a decrease in trabecular number. The result was no net change in distal femoral BV/TV.

**Figure 5.**
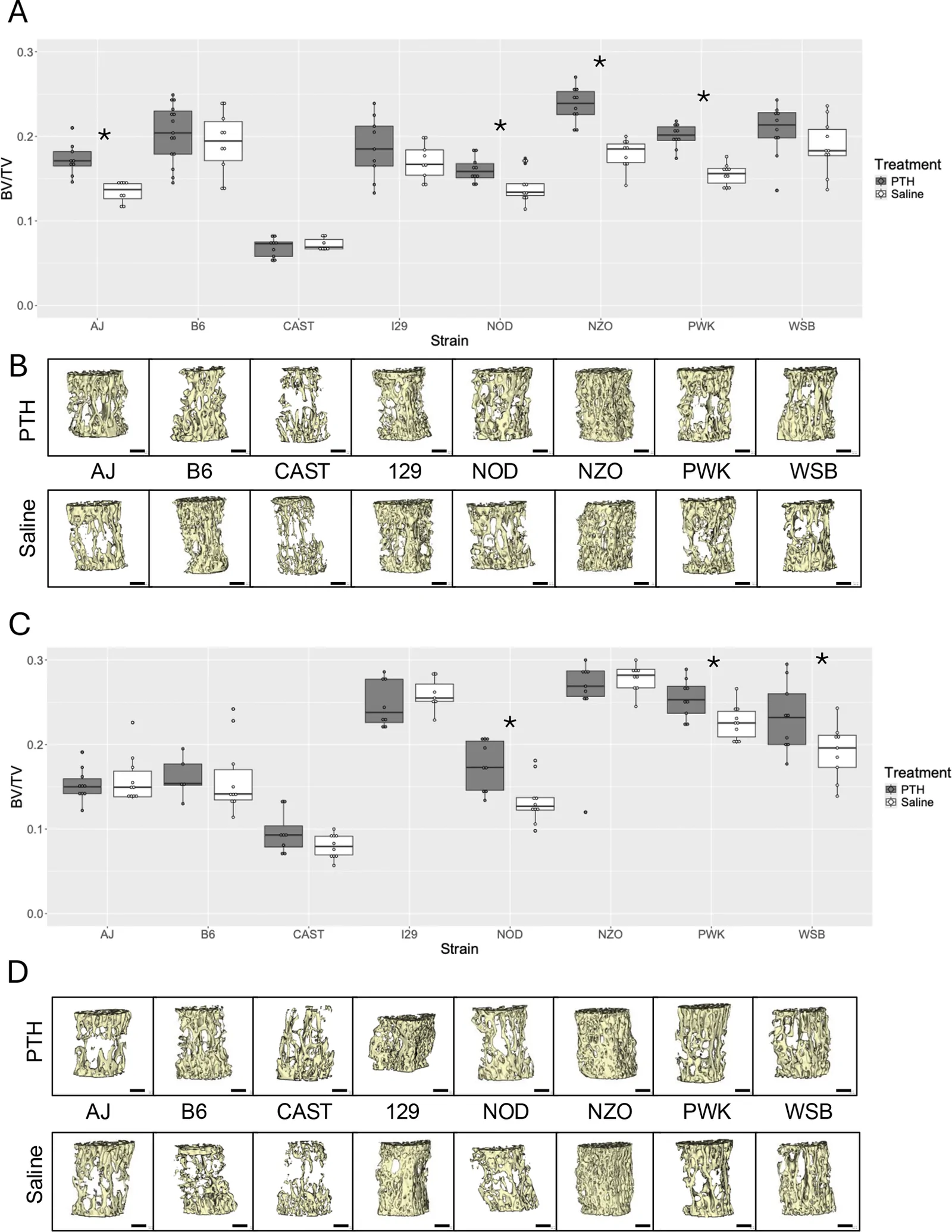
Lumbar vertebral trabecular bone volume fraction (BV/TV) in saline and PTH treated mice. Volume fraction (BV/TV) of the fourth lumbar vertebrae was measured by μCT after 4 weeks of PTH treatment. Date from the PTH treated (grey) and saline treated (white) males (A) and females (C) is present. Representative reconstructions of the trabecular compartment are shown for males (B) and females (D) such that the PTH treated animals are on the top row and the saline treated are on the bottom row. *p<0.05.

In the strains that experienced a response in vertebral trabecular bone volume (BV/TV), change was always in the positive direction in the males (Figure 5A & B and **Supplemental Table 6A)**. As occurred in the distal femur, a response was seen in both the A/J and NOD males. A substantial increase in BV/TV with PTH was seen in the NZO and PWK males as well. For all four strains, this increase in BV/TV corresponded to an increase in trabecular thickness, but unlike in the distal femur, no change in trabecular number was associated with this increase. As was observed in the femur, trabecular thickness increased in the vertebral body, but trabecular number decreased in the B6 males, with no net change in BV/TV. There was sufficient trabecular bone in the vertebral body in the CAST males to measure trabecular number. In response to PTH, this value strongly decreased in this strain, but this was not associated with any change in BV/TV.

A very different group of strains presented with a change in BV/TV after PTH treatment in the females, compared to the males, for the distal femur (Figure 4C & D and **Supplemental Table 5B)**. Here, BV/TV increased in the 129 and the WSB. In the 129, this was associated with an increase in trabecular thickness and no change in trabecular number. Conversely, in the WSB there was no change in trabecular thickness but there was a dramatic increase in trabecular number. However, as occurred for the males, CAST females experienced a decrease in distal femoral BV/TV with PTH treatment. Unlike in the males, this was not associated with any change in trabecular thickness, and trabecular number could not be measured. Increases in trabecular thickness were also observed in the NOD, NZO and PWK but this was not associated with any change in BV/TV. In the vertebral body BV/TV increased in the NOD, PWK and WSB in the females treated with PTH (Figure 5C & D and **Supplemental Table 6B)**. For both the NOD and the PWK this increase was accomplished by increasing trabecular thickness, with no associated change in trabecular number. In the WSB females, PTH treatment did not result in a statistically significant change in either trabecular thickness or trabecular number despite the substantial increase in BV/TV. Overall, the response to PTH was most heritable for BV/TV at both the distal femur and in the vertebral body (Table 1).

## DISCUSSION

The eight strains of mice used in this study were selected as they are the founder strains for a very powerful genetic mapping population called the Diversity Outbred ^(13)^. This resource is increasingly becoming the preferred outbred mouse model for genetic mapping studies in mice. The genomes of these 8 strains have been sequenced and among these strains there are ~37.8 million single nucleotide polymorphism (SNP) differences and ~6.1 million insertion/deletions (INDELs) ^(14)^. In comparison, the 1000 genomes project has suggested that only 2.7 million SNPs appear in humans with a minor allele frequency of greater than 1% ^(15)^. These eight strains represent a considerable amount of genetic variability, allowing for testing of heritability of the response to teriparatide/PTH in a relatively small number of mice. There are many advantages to studying drug response heritability in mice compared to humans. In mice, the environment is tightly controlled, including temperature exposure, light/dark cycle, and diet. There are no concerns for complicating factors such as medication use or differences in activity levels. Moreover, all mice within an inbred strain can be considered genetic clones of all other mice of that strain ^(16)^, allowing for repeated measures of phenotypes to overcome subtle differences that could be masked due to technical or measurement error. Further, by using mice we can collect data that cannot be collected in human cohorts such as bone strength and stiffness.

As more data is accumulated, it is becoming clearer that treatment failure and inadequate clinical response after teriparatide therapy is a significant problem for patients. A retrospective analysis of clinical trials data has shown that dose and duration of treatment matter when considering changes in BMD ^(6)^. A limitation of that analysis was that the clinical trial data were available only for women. At 12 months of dosing with 20ug/day, the retrospective analysis showed that 13% of women did not attain a 3% or more increase in BMD at the lumbar spine and 68% did not attain a similar increase in BMD at the hip. At 18 months, the non-responder rate based on BMD ranged from 6 to 9% for the lumbar spine and 50 to 53% for the hip. At a higher dose of 40ug/day only 6% of patients did not respond at the lumbar spine. Available bone turnover markers such as P1NP were not able to explain why patients did not respond ^(6)^; however, other studies have suggested that non-responders to teriparatide tend to demonstrate lower levels of bone turnover at treatment initiation ^(17)^. Cohort data is also available that corroborates the observations seen in clinical trials. A small prospective study of post-menopausal women found a non-response rate for lumbar spine BMD of 15%, and like what was observed in clinical trial data, the non-response rate for hip BMD was much higher at 55% ^(7)^. These rates of non-response are similar to that observed in retrospective studies of teriparatide response ^(17,18)^. In our data, half of the strains responded in both males and females for whole body BMD, but there were differences in which strains responded between the two sexes. Our data strongly suggest that response to PTH is genetic and sex specific, but a study in outbred mice will be needed to test more complex allelic distributions and to determine the genes responsible for such a genetic response. As in humans, we found that some strains were very resistant to PTH. WSB males did not respond for any of the phenotypes we measured and A/J females only showed a response for the phenotypes of intramedullary area and cortical thickness.

Fracture after treatment with teriparatide remains an issue for some patients. In a retrospective study of men and women, Elraiyah et al found that up to 7% of patients continued to fracture after at least 6 months of teriparatide treatment ^(8)^. In this cohort of men and women, 34.8% of patients failed to achieve a 3% or greater increase in BMD at the spine, hip, or both sites. In a retrospective study of osteoporotic treatment effectiveness in post-menopausal women (the TAILOR study), an 11% treatment failure rate was observed in patients treated with teriparatide ^(19)^. In that study, failure was defined as two or more incident fractures after the initiation of treatment ^(20)^. In our mouse study, the heritability for increased femur flexural strength after PTH treatment was 56% in males and 49.2% females (Table 1), indicating that half of the variation in response to PTH for femur strength can be attributed to mouse strain, which in our study equates to genetic factors. Based on our mouse femur data, we postulated that just as occurred for changes in BMD ^(11)^, fracture after teriparatide treatment can in part be explained by a patient’s genetics. It is not practical to collect such data in humans, as heritability of fracture is very difficult to estimate, and the data do not exist in large enough numbers to provide for matched treated and untreated cohorts for fracture ^(21)^. Our data showed that increased femur strength at mid-diaphysis coincided with an increase in the amount of cortical bone. The mechanism by which bone strength is changed is very different in mice versus humans, as teriparatide treatment was not associated with an increase in cortical thickness in either pre-menopausal ^(22)^ or post menopausal women ^(23)^.

A general practice for studies investigating teriparatide response has been to compare studies in men to literature reported data collected in women ^(24)^. However, in a single retrospective study of men and women that had completed at least 12 months of teriparatide therapy, Niimi *et al* showed that there was no difference between men and women for the absolute increase in lumbar spine BMD and femoral neck BMD, but longitudinally their data suggested that women were more responsive than men at the femoral neck at earlier timepoints ^(25)^. In a small study of eugonadal men and post-menopausal women, it was observed that women, but not men, would lose BMD at the distal radius during active treatment, and women would mount a larger P1NP response than men. However, upon cessation of treatment women lost more bone than men ^(26)^. Non-response rates to teriparatide appear similar in men and women ^(27)^. Our study was able to directly compare males and females within and between different genetic backgrounds, highlighting important sex-specific responses. For example, in all strains that showed a cortical response to PTH, treatment induced an increase in cortical area but the mechanism by which this happened was different among the strains and by sex. Regardless of sex, the increase in cortical area was largely associated with an increase in cortical thickness. In male mice, intramedullary area did not change except in the CAST mice. This suggests that in male mice cortical bone mass was most commonly increased largely by periosteal expansion. In female mice a decrease in intramedullary area was more common, suggesting a trend towards endosteal expansion instead. These mice were 12 weeks of age at the initiation of treatment. By definition, all of these mice could be considered sexually mature, but mice at this age are still actively growing ^(28)^. This male/female difference in periosteal expansion is highly reminiscent of that seen in humans surrounding puberty and with aging ^(29)^. There were clear deviations to this trend in select strains. Specifically, CAST males and females presented with decreased intramedullary area and neither PWK males nor females showed any significant changes in intramedullary area. This suggests that while there are sex-specific patterns, genetic factors may be able to override them.

Multiple clinical studies have shown that the skeletal anabolic response to intermittent teriparatide is stronger at the spine than at the hip ^(6)^. This is reflected in fracture data which show that regardless of dose, teriparatide is more effective at reducing vertebral fractures than non-vertebral fractures^(30)^. In randomized control studies the overlap between non-responders at the spine and hip was not perfect ^(6)^. In other words, some patients responded at both sites whereas some patients responded at the hip but not the spine and vice versa. We saw a similar phenomenon in our data. For the males, only one strain (NOD) showed a change in trabecular bone volume (BV/TV) in both lumbar vertebrae and the distal femur. In females, there was no overlap in response to PTH for trabecular bone in the lumbar spine and femur among the strains that responded to treatment. This finding is reflected in genome wide association study (GWAS) data that show that there are many loci that impact BMD only at select anatomic sites ^(31)^. This is further supported in studies showing that osteoblast gene expression and mineralizing ability is different for cells taken from different anatomic sites ^(32)^.

We saw a decrease in BV/TV in the femur in male B6, CAST and NOD mice and in CAST female mice. A similar phenomenon was not seen in the vertebra. Studies of single doses of teriparatide showed that resorption is immediately increased after dosing, before formation begins to increase ^(33)^. The kinetics of turnover in different mouse strains is poorly understood, but it is known that osteoblast mediated formation and osteoclast number are both independently genetically controlled ^(34,35)^. It was not possible to measure serum bone turnover markers in all strains of mice as the wild derived mice are very small, precluding safe blood draws. We hypothesize, based on known differences in inbred strains and transgenic mouse models, that some strains were more sensitive to the PTH with regards to ramping up bone resorption in the femur. This same reaction to teriparatide, however, may happen in humans. Using HR-pQCT, a decrease in BV/TV was observed at the distal radius and the distal tibia in women after 18 months of teriparatide therapy. The authors of this study speculate that teriparatide resulted in an increase in number of trabeculae, albeit each one smaller and thinner in size ^(36)^, but this is not what we observed in mice. In the male mice that experienced a decrease in BV/TV, trabecular number also decreased. We did observe that an increase in trabecular number could be associated with an increase in osteoclast number in some transgenic mice ^(34)^.

## CONCLUSIONS

As this study of mice has repeated measures within a given genotype, we can conclusively show that there are non-responders to PTH treatment, but our data clearly show that response is heavily driven by genetic factors. Further, these data suggest that a primary driver of skeletal response to PTH stems from interactions between genetics and sex. This work supports the future use of outbred mice to conduct GWAS studies for the response of bone to intermittent PTH to determine the genes responsible for mediating the effect of intermittent PTH on bone.

## Supplementary Material

Supplemental Table 1: Body weight and composition for males (A) and females (B).

Supplemental Table 2: Whole body BMD and BMC for males (A) and females (B) as measured by DXA.

Supplemental Table 3: Femoral cortical architecture as measured by μCT for males (A) and females (B)

Supplemental Table 4: Femoral cortical mechanical properties as measured by 3-point bending for males (A) and females (B)

Supplemental Table 5: Femoral distal trabecular architecture as measured by μCT for males (A) and females (B)

Supplemental Table 6: Vertebral (L4) trabecular architecture as measured by μCT for males (A) and females (B)

## Figures and Tables

**Table 1.** Broad sense heritablities (H^2^, %) for response to PTH

Phenotype	Male	Female
BMD	38.9	85.0
Cortical Area	71.0	39.1
Breaking Strength	56.0	49.7
BV/TV (femoral)	64.8	87.8
BV/TV (vertebral)	77.2	76.1

**Table 2.** Summary of the impact of PTH by sex, bone compartment and anatomic location.

Strain	Sex	Whole body BMD	Cortical Area	Breaking Strength	Femoral BV/TV	Vertebral BV/TV

129	Male					
	
	Female	↑	↑		↑	

A/J	Male	↑	↑	↑	↑	↑
	
	Female					

B6	Male	↑				
	
	Female	↑	↑	↑		

CAST	Male		↑		↓	
	
	Female				↓	

NOD	Male	↑			↓	↑
	
	Female	↑	↑	↑		↑

NZO	Male					↑
	
	Female					

PWK	Male	↑	↑	↑		↑
	
	Female	↑	↑			↑

WSB	Male					
	
	Female				↑	↑

## Data Availability

All raw phenotyping data is available upon request. No specialized code was generated for the data analysis, and all analysis was conducted using the open-source packages for R listed in the methods.
